# Machine learning approach for study on subway passenger flow

**DOI:** 10.1038/s41598-022-06767-7

**Published:** 2022-02-17

**Authors:** Yujin Park, Yoonhee Choi, Kyongwon Kim, Jae Keun Yoo

**Affiliations:** Department of Statistics, Ewha Womans University, Seoul, 03760 South Korea

**Keywords:** Applied mathematics, Scientific data, Statistics, Civil engineering

## Abstract

We investigate regional features nearby the subway station using the clustering method called the funFEM and propose a two-step procedure to predict a subway passenger transport flow by incorporating the geographical information from the cluster analysis to functional time series prediction. A massive smart card transaction dataset is used to analyze the daily number of passengers for each station in Seoul Metro. First, we cluster the stations into six categories with respect to their patterns of passenger transport. Then, we forecast the daily number of passengers with respect to each cluster. By comparing our predicted results with the actual number of passengers, we demonstrate the predicted number of passengers based on the clustering results is more accurate in contrast to the result without considering the regional properties. The result from our data-driven approach can be applied to improve the subway service plan and relieve infectious diseases as we can reduce the congestion by controlling train intervals based on the passenger flow. Furthermore, the prediction result can be utilized to plan a ‘smart city’ which seeks shorter commuting time, comfortable ridership, and environmental sustainability.

## Introduction

There has been a momentous development of real time data collecting technique from the smart card system which can make us possible to track subway passenger’s travel patterns. After the outbreak of Covid-19, analyzing the regional characteristics and predicting the number of commuters and travelers in the subway has gained a great attention as controlling subway intervals can reduce the density of passengers, which can mitigate the spread of the virus in the subway. In the long run, an improved operation plan based on the geographic information nearby the station and passenger transport flow can enhance the ridership experience because more citizens are willing to take a subway instead of a vehicle. Subway passenger management using big data analysis can support urban planning as policymakers can make a data-driven decision for the locations of the subway stations for the ‘smart city’. The Seoul metropolitan government of Korea is developing urban plans to expand the railway network to establish a sustainable transportation system, develop an eco-friendly subway operation plan, provide real-time congestion information to reduce the spreading of COVID-19, and offer a comfortable subway ridership experience to passengers. Seoul also put multilateral efforts into building an energy-efficient city.

We utilize a functional data analysis (FDA) approach to the Seoul Metro subway dataset to cluster the stations based on their geographical feature and predict passengers flow. In particular, we have two main contributions. First, we use a recently developed model-based functional clustering analysis method to characterize the regional features nearby the stations into six categories, such as residential, business, and commercial areas. Functional clustering can successfully capture geographical characteristics. After we categorize the subway stations, we predict the passenger transport flow with respect to each cluster. We further compare the prediction accuracy of our method to the case of when we predict the number of passengers without considering regional characteristics. We demonstrate the information from the clustering analysis can significantly improve the accuracy. To our best knowledge, there has not been any study about applying clustering analysis outcomes to predict the number of subway passengers. Our results also have several contributions to society. First, by adjusting the subway operation plan based on the more accurately predicted passenger transport flow, we can prevent congestion in the subway, which is helpful for the better passenger ridership experience and refraining from the transfer of an infectious disease. Furthermore, our research is helpful for urban smart city planning as we can decide the location of the subway stations to reduce the chance of overcrowding by considering the geographical attributes from the clustering analysis. This can provide a comfortable ridership experience to passengers and lead them to use public transportation, which can be helpful for many environmental issues.

The rest of the paper is organized as follows. In “Related work” section, we investigate some related research about subway passengers and urban transportation planning. “Result” section presents the results of the data analysis. We provide some discussions in “Discussion” section. “Methods” section describes the theoretical background of FDA, functional clustering, and functional time series prediction methods. We put some additional numerical results in Appendix.

## Related work

A variety of studies have been conducted to analyze and predict the number of subway passengers. Tang et al. and Wang et al.^1,2^ introduced a semantic method to identify spatio-temporal latent functions of subway stations in Shanghai, China, based on the mobility patterns. They utilize the smart card transaction data, network data, and point of interest information of each station. They especially cluster the stations in ten functional clusters and present latent functions of them. Ling et al.^3^ compared the performance of the historical average model, neural network model using multilayer perceptron, support vector regression model, and gradient boosted regression trees model to predict the dynamical passenger flow. Kim et al.^4^ analyzed the space-time variability of subway passengers data in Seoul using cyclostationary empirical orthogonal function. Similar to this, Yu et al.^5^ utilized smart card data of Nanjing Metro, China, to find a commuting characteristic of residents. Shin^6^ applied a fluid dynamic model to Seoul metropolitan’s big data to extract commuting patterns. An additional method was presented to predict subway passenger flow using the particle swarm optimization algorithm^7^, which incorporates backpropagation in a neural network with empirical mode decomposition. Cluster analysis of the New York city dataset was conducted by^8^. Based on the server, Alan and Birant^9^ proposed a personalized fare calculation system to provide benefits to passengers who do not have a specific smart card for regional transportation. For a more comprehensive review of analyzing subway datasets, see^10^.

Furthermore, there has been some research about urban transportation planning. For example, Lim^11^ presented a comparative analysis between the urban design policy in Seoul and the transit-oriented development policy of Singapore and Tokyo to support the idea of transforming Seoul into a transit-friendly city. Oh et al.^12^ introduced the model to analyze important latent factors in the supply and demand of the number of subway passengers. Because the regional attributes can be regarded as a piece of useful information to predict passenger transport, there was research about their relationship. For example, Sohn and Kim^13^ investigated the relationship between the number of passengers and the regional characteristic of the urban transit stations in the Seoul metropolitan area. Similar to this, Lee et al.^14^ categorized the regional property of the nearby subway stations and implemented correlation analysis between the regional characteristic and passenger flow patterns. Kim et al.^15^ further implemented cluster analysis by using the information from the ratio of land usage and the distance between subway stations. Sung and Kim^16^ applied a multidimensional scaling method to factor analysis to investigate the regional characteristic and passenger flow. Choi et al.^17^ and Lee et al.^18^ focus on the relationship between the characteristics of stations and passenger patterns.

## Result

Seoul is known as one of the largest metropolitan cities in the world, with more than 310 subway stations from 10 lines. The number of passengers for each line is approximately 700,000 and 300,000 during the weekdays and weekends, respectively. In this study, the dataset we are interested in is the smart card transaction data tracking the number of passengers from January 2020 to December 2020 in a 1-h interval using 224 subway stations in Seoul Metro from lines 1 to 8. Seoul metropolitan government provides a massive smart card transaction dataset and we obtain the dataset from https://data.seoul.go.kr.

### Explanatory data analysis

**Figure 1:**
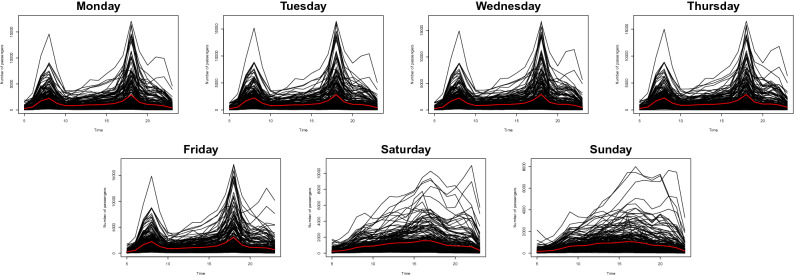
The pattern of the averaged daily number of passengers entering the subway station for 224 stations from January 6, 2020, to January 12, 2020 (black lines) with their sample mean (red lines).

For each station, we split our dataset into the number of passengers who get on and off the subway. To obtain some insights, we visualize the daily number of passengers who enter the subway station from Monday to Sunday in Fig. 1. To see the daily passenger flow patterns, we plot the number of passengers entering the stations in a 1-h interval for each day of the week, from January 6, 2020, to January 12, 2020. As we can see from the first five plots in Fig. 1, in the weekdays, we can observe there are two peaks; the first is around 7 to 8 in the morning and the second one is 6 to 7 in the afternoon. This aligns with our intuition as both peaks present the commuting hours. The sixth and seventh figures of Fig. 1 indicate the number of passenger transport on the weekends. We cannot see any peaks in the commuting time because not many people go to work during the weekends. However, we notice there are more passengers on Saturday than Sunday. The patterns of passenger flow on the weekends are moderate than the weekdays, and the maximum number of passengers during the weekends is around half of the weekdays. Moreover, Fig. 1 indicates the difference in the number of passengers between the stations is amplified on the weekdays.

**Figure 2:**
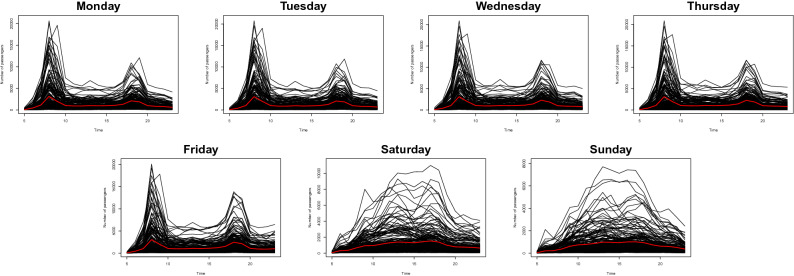
The pattern of the averaged daily number of passengers leaving the subway station for 224 stations from January 6, 2020, to January 12, 2020 (black lines) with their sample mean (red lines).

Figure 2 represents the number of passengers who get out of the subway during the week. In general, they show similar patterns to Fig. 1. One thing that we can notice is that Sillim station has the highest number of passengers who get on the train around 7 to 8 A.M. and get off the subway station around 6 to 7 P.M. This result is well aligned with our intuition because the area near Sillim station is famous for being one of the biggest transit centers in Seoul. People who live in the suburban part of Seoul utilize this station to transfer. On the other hand, Gangnam station shows the opposite pattern to Sillim station as it has the largest number of passengers who get on the train around 6 to 7 in the evening and leave the station around 7 to 8 in the morning. Again, this result is well aligned with our intuition in that the region near the Gangnam station is one of the largest business and commercial areas in Seoul. Moreover, we can observe that the number of passengers from Monday to Friday and Saturday to Sunday shows a similar pattern, respectively, and the patterns of the number of users on weekdays and weekends present a significant difference. Therefore, we organize our dataset into four categories; that is, the number of passengers who get on the subway during the weekdays (DN), get off the train during the weekdays (DF), get on the subway during the weekends (KN) and get off the train during the weekends (KF).

**Figure 3:**
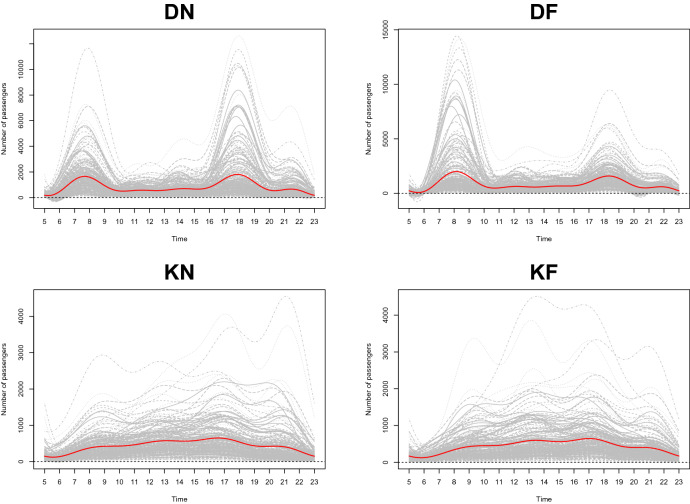
Smoothed curves of the averaged daily number of passengers for four situations in a 1-h interval with Fourier basis (gray lines) and their sample mean functions (red lines).

We use the FDA approach to capture the subway pattern based on the shape of the observed functions. First of all, because we only can observe a finite number of data points, we convert our dataset into a functional form using the Fourier basis with the number of bases is 10 under the assumption that our observed dataset is an independent realization of $L_{\scriptscriptstyle {2}}$ stochastic process. Figure 3 presents the smoothed 224 curves on the interval (5, 24) for four categories, DN, DF, KN, and KF, with respect to Fourier basis expansion. We try a variety of the number of bases, and we decide to use 10 because it can reasonably smooth the functions and retain the dataset’s characteristics simultaneously. The red lines in Fig. 3 represent the sample mean function. They show similar patterns to the original time series means in Figs. 1 and 2.

**Figure 4:**
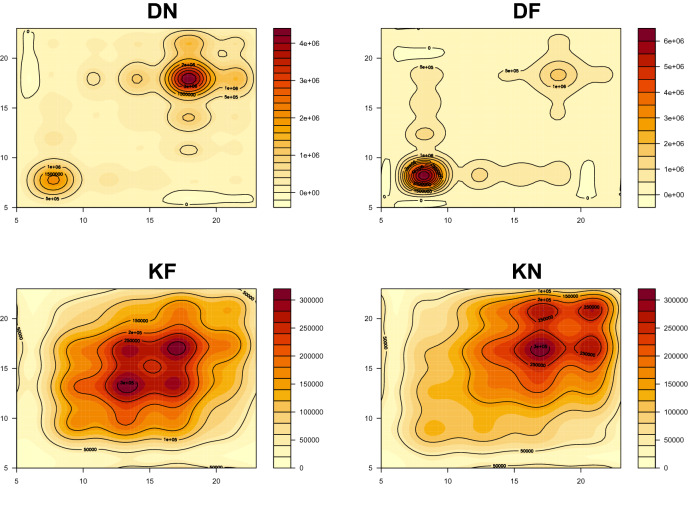
The contour plot of the covariance functions for the daily number of passengers in four situations.

Figure 4 presents the surface of the covariance functions. As we can see from the first plot in Fig. 4, the DN case shows the highest variance around 6 P.M. This is because a few stations at 6 P.M. have a large number of passengers for commute while a majority of stations have a moderate number of people at this time because the business districts tend to be concentrated in specific areas of Seoul. On the other hand, the covariance function of DF has the highest value around 8 A.M. This is also because a few stations in the business district have a large number of passengers who get off the subway to head to their workplace around this time. For KN and KF, covariance functions have an increasing trend toward the evening. This is also intuitive as not many people use a subway for their commute during the weekends. However, passengers are likely to use the subway more in the afternoon for their personal life.

**Figure 5:**
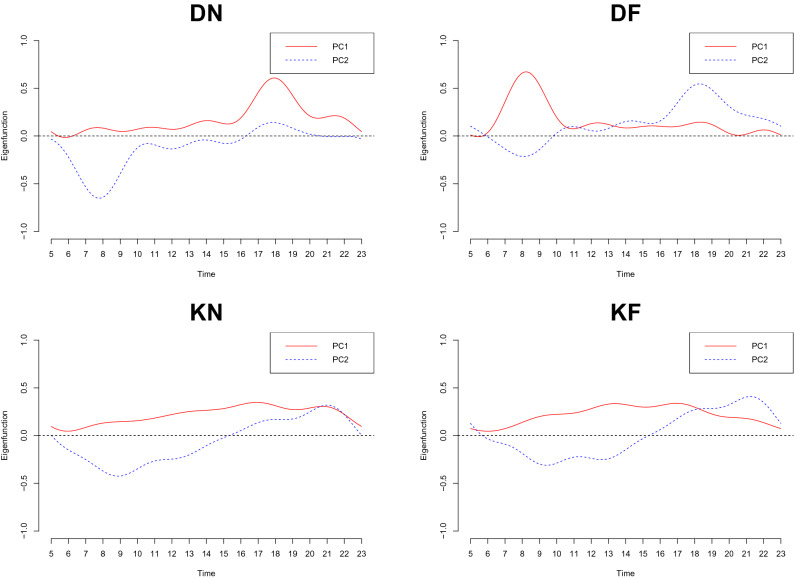
The first two eigenfunctions from the PCA result for four scenarios.

To investigate the main variation in daily passenger flow, we apply FPCA to our dataset. The red solid lines in Fig. 5 present the first principal component and the blue dotted lines are for the second principal component. For the passenger flow of getting on the train in the morning, the first two principal components explain $96.4\%$ of the variability around the mean function. As we can notice from the first plot of Fig. 5, the first principal component shows a positive impact on the mean function around 6 to 7 P.M. The highest score station of this type of pattern is for the stations in the commercial or business areas as they have a larger passenger flow who get on the train in the afternoon. The second principal component of DN shows a negative variation near the mean function in the morning. The station with the highest score in this situation is also a business and commercial district because some stations in these areas tend to have a small number of passengers who get on the train in the morning. For DF situation, the first two principal components account for $96.6 \%$ of variability. For the first principal component, there is a strong positive impact on the mean curve in the morning. This also well aligns with our intuition and the commercial areas take the highest score for the first principal component as many commuters leave the station in the commercial or business district in the morning. The second principal component of DF has a negative and positive effect on the mean in the morning and evening, respectively. The situations with the higher scores are residential areas because in these areas, not many people leave the stations and a majority of passengers get off the train in the afternoon. For the weekends, the first two principal components for the passengers who get on and off the train account for $97.3\%$ and $96.8\%$, respectively. Furthermore, their principal components show a similar pattern as the first principal component presents an increasing positive variability toward the evening hours and it has a peak around 5 to 6 P.M. The second principal component shows a negative impact on the mean curve near 9 to 11 A.M. In particular, for KN, the highest score for the second principal component goes to the commercial areas as there is a limited number of passengers in the morning and a larger number of people in the evening as family goes out to enjoy the weekends around this time. With the symmetric reason, the areas with the highest score for the second principal component in the KF case can be regarded as residential areas.

The numerical results in this paper are implemented using an Intel Core i7 CPU with a 2.90 GHz processor. Table 1 shows the computing time of FPCA.

**Table 1:** The execution time for FPCA in seconds.

DN	DF	KN	KF
0.018	0.011	0.024	0.018

### Functional clustering analysis results

To categorize the stations based on their passenger flow characteristics, we use the functional clustering method called the funFEM^19^. The averaged daily passenger transport data of 2020 in a 1-h interval is used. We incorporate DN, DF, KN, KF in parallel to consider the patterns of these four situations simultaneously. We have tried clustering based on a whole daily data, seasonal averaged data, and annually-averaged settings. We report the result of daily averaged data because it can capture the regional characteristics nearby subway stations efficiently. We also consider another functional clustering method called funHDDC^19,20^ algorithm. However, we only present the results from the funFEM as the outcomes from the funFEM are more interpretable and well aligned with our intuition. The results of our clustering analysis have their advantage in finding particular shape patterns for each cluster, not just categorize based on the size of the functions. Furthermore, the functional clustering analysis takes only 4.680 s.

**Figure 6:**
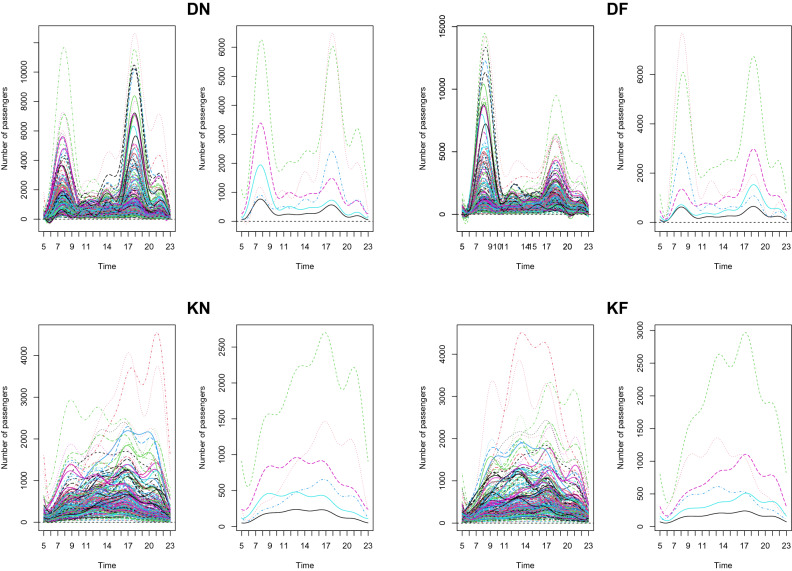
The results of the clustering analysis for all 224 stations (left) and the mean function for each cluster (right) in four cases.

Figure 6 presents the result of cluster analysis for four situations. For each scenario, we provide the whole functional patterns on the left-hand side and their mean functions with respect to six clusters on the right-hand side. Cluster 1 is expressed in a black solid line. The stations in this cluster only have a relatively small peak in the morning for the situation of DN and show a moderate pattern with small passenger flows throughout the day. The regional property near these stations can be regarded as an area which is dominated by a non-working age group, less than 15 years old or more than 65 years old. Furthermore, these regions are not considered as commercial or business areas. Green dotted lines represent the second cluster. The stations in this category have their peaks in the afternoon for all cases. We also can note that the highest number of passengers who enter the station is smaller than its value for the passengers who leave the station. Therefore, the regional characteristics near these areas can be regarded as downtown commercial areas or transit centers. The weekend patterns of this cluster show a similar shape to its weekday counterpart. Finally, we also can see the cluster 2 has a higher volume of passengers compared to all other clusters throughout the situations. Red dotted lines in Fig. 6 indicate the third cluster. This cluster shows the peak number of passengers who get on the train around 5 to 6 P.M. and get off the subway around 7 to 9 A.M. during the weekdays. Furthermore, the volume of passenger flows during the weekends is significantly lower than that of weekdays. From these passenger transport patterns, the regional characteristics of nearby stations in cluster 3 can be considered as a business district such as Gasan digital complex, Seolleung, Express bus terminal. Blue dotted lines in Fig. 6 represent the cluster 4. The stations in cluster 4 have similar passenger flow patterns to the stations in cluster 3. However, the size of passenger transport in cluster 4 is significantly smaller than the cluster 3. Thus, the regional property of stations in cluster 4 can be considered as relatively small business areas. Pink dashed lines in Fig. 6 indicate the cluster 5 and they show the opposite property to red dotted lines. The lines in cluster 5 have their peak of DN in the morning and DF in the afternoon. This means passengers are more likely to enter the station around 7 to 8 A.M. and leave the station around 6 P.M. Based on this characteristic, a residential district can be appropriate to explain the regions nearby the stations of cluster 5. Light blue solid lines present the 6th cluster. As we can see from Fig. 6, they have a similar shape to lines of cluster 5. However, the size of the functions in cluster 6 is smaller than cluster 5. Therefore, we can decide the regions near the stations of cluster 6 are small size residential areas. We also can note that there are small spikes for the clusters 5 and 6 in the number of passengers who are leaving the stations during the weekends. This is well aligned with our intuition because it is expected that there is a large number of passenger transport who head back to their home in the evening of the weekends.

**Figure 7:**
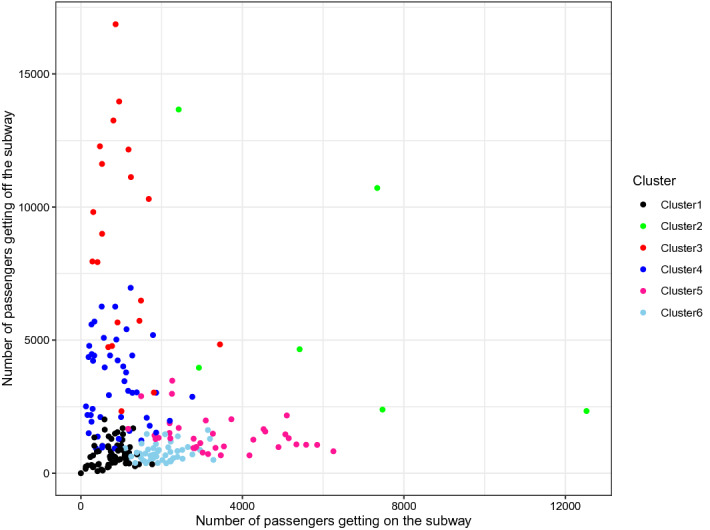
Scatter plot of the number of passengers get on and off the subway in 8 A.M.

**Figure 8:**
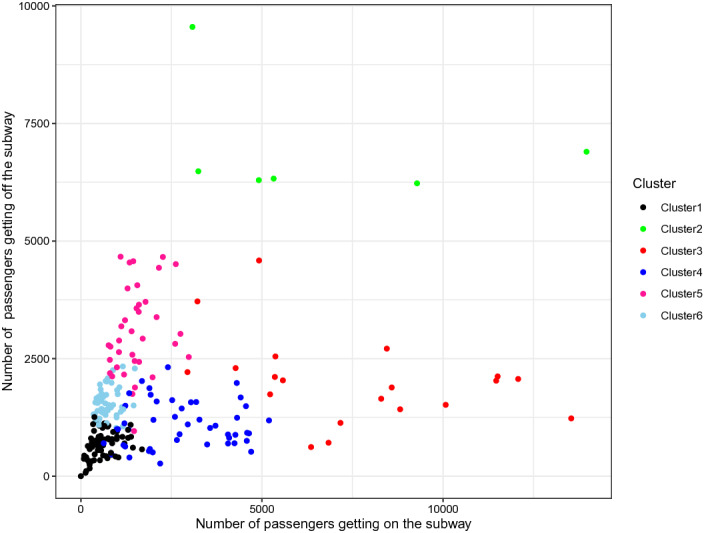
Scatter plot of the number of passengers get on and off the subway in 6 P.M.

To better illustrate the characteristics of each cluster, we present scatter plots which have the number of passengers who get on the train in *X*-axis and the number of people who get off the train as *Y*-axis. Figure 7 represents the dataset from 8 A.M., and Fig. 8 indicates the situation of 6 P.M. We can notice that clusters 5 and 6 indicate the stations near the residential area as a large number of passengers enter the station in the morning compared to the number of passengers who get off the train, and we can see the opposite trend in the afternoon. We can also clearly see that the clusters 3 and 4 stand for the stations in the business district because we can see the opposite patterns to clusters 5 and 6. In general, the stations in cluster 2 have a higher number of passengers than the other clusters. Thus, the regional properties nearby the stations of cluster 2 present a downtown or transit centers. Cluster 1 is located in the opposite direction to cluster 2, and the stations in cluster 1 have a relatively lower volume of passengers transport. Therefore, we regard the regional property of stations near cluster 1 as the areas with a fewer number of working-aged people. Finally, we can notice that the left and right plots are symmetric, which is also well aligned with our intuition.

**Figure 9:**
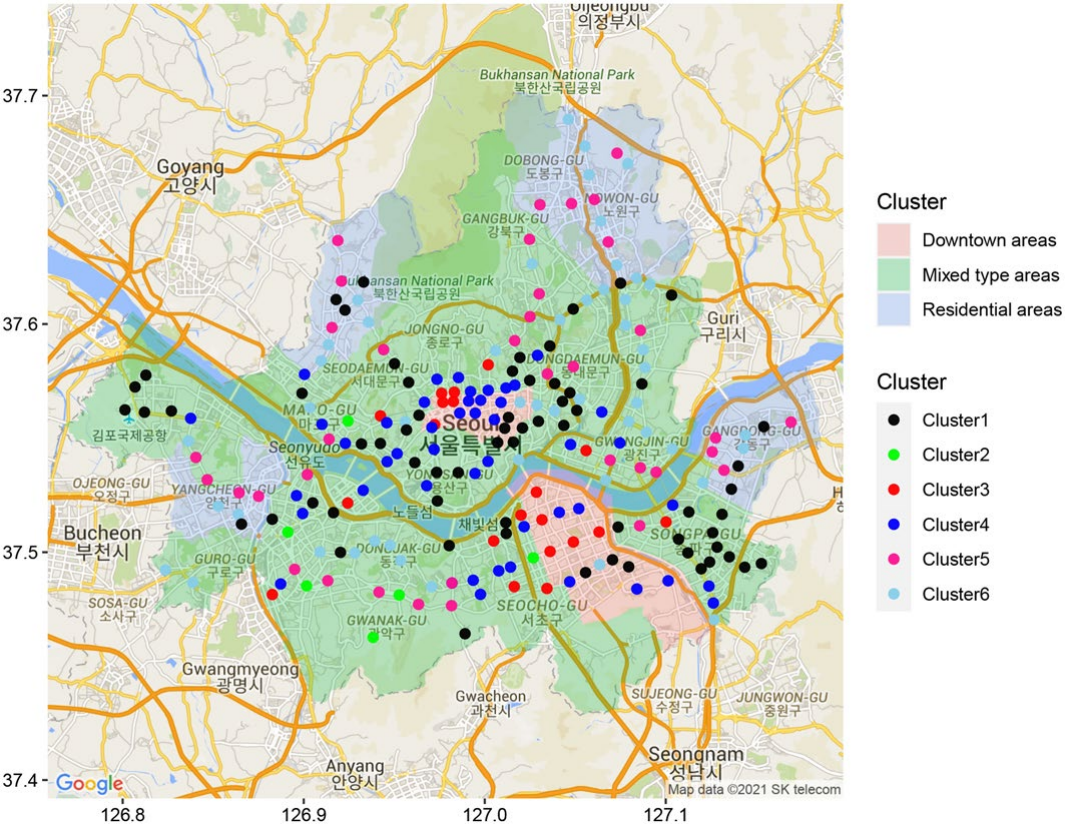
The location of subway stations in Seoul based on the results of the clustering analysis. (Map data is from Google and SK telecom. This map is annotated using *ggmap* package (https://cran.r-project.org/web/packages/ggmap/index.html) in R 4.0.5^21^).

Figure 9 presents the location of 224 stations in Seoul. We color every station with respect to their clustering result. We can see that the stations in categories 3 and 4 are concentrated in the middle of the city, such as Gangnam and Jung districts. On the other hand, clusters 5 and 6, which include the stations in the residential region, are located outside of the center. We can also see that the business district is surrounded by residential areas. This result is intuitive as the policymakers tend to construct residential areas around the business district so that people can enjoy a shorter commuting time. In Fig. 9, we divide the districts in Seoul into three categories; that is, downtown, residential, and mixed type areas which are shaded in red, blue, and green, respectively. Among the blue shaded districts, the northeast areas are Nowon and Dobong district, the northwest part is the Eunpyeoung district, the southeast region is the Gangdong district, and the southwest sector is the Yangcheon district. We can observe that the stations in the residential area in the southwestern part of Seoul are dominated by the stations in cluster 5. On the other hand, we can observe that the other residential areas have stations in clusters 1, 5, and 6. This is noticeable as the populations of the four residential areas are similar. Seoul metropolitan government has intentionally developed these areas to relieve housing problems. However, the Yangcheon district still needs more subway stations because all of the subway stations in this area are in cluster 5, which means they have a high volume of passenger transport flow. In fact, the stations in this area are notorious for a large number of complaints regarding the congestion. Policymakers can allocate more resources for a better ridership experience by providing more public transportation infrastructure in this area. Therefore, we can see our clustering analysis can capture the city’s characteristics, reflect the city development plan, and be utilized in future ‘smart city’ development.

**Figure 10:**
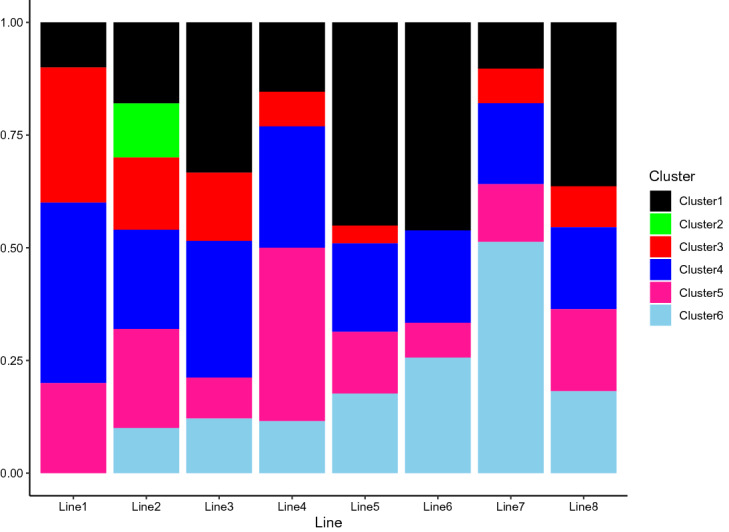
The composition ratio of clusters by subway lines.

Figure 10 presents ratios of the number of stations for each cluster by lines. The Seoul metropolitan government names the line numbers in order; that is, line 1 is the oldest, and line 8 is the most recently developed subway lines. The line number 1 was created in 1974, and almost all stations are in the clusters 3 and 4, business or commercial areas. This is because South Korea was one of the fastest developing countries in the world in 1975 and they have a great demand of passenger transport nearby working spaces. The second subway line is developed in 1978. Line 2 includes stations of cluster 2, transit center or downtown, and we can see a few stations are in clusters 5 and 6, residential areas. This reflects the history that South Korea planned to develop the southern part of Seoul in 1980’s. To disperse people who live in the northern part of the Han river, the government developed residential areas with transit centers in the southern part of the Han river to induce people to move to the south. Line number 2 plays a critical role in satisfying the increasing demand for public transportation in this area. It is known that line 2 takes more than $50\%$ of complaints in Seoul Metro. This could hinge on a large number of passengers, especially for the stations in cluster 2. Developing an express train system for these stations or shortening the train interval in peak hours can relieve the high-density problem. Regarding the line number 3, we can see that the proportions of clusters 3 and 4, and clusters 5 and 6 are almost identical. Furthermore, we can see there are more stations in residential areas clusters than clusters 3 and 4, for lines 4, 5, 6, 7,  and 8. Since the economy was expanding very fast in 1980’s, there was great housing demand in Seoul. Therefore, the government focuses on the development of residential areas and provides infrastructure in these areas to accommodate more people. Finally, we insert the clustering result of all 224 stations in Seoul, which can be found in Table 2.

**Table 2:** The list of stations for six clusters.

Cluster	Station
1	Garak Market, Gyeryong, Gaehwasan, Geoyeo, National Police Hospital, Godeok, Korea University, Gwangheungchang, Gusan, Geumho, Gildong, Gimpo Airport, Namtaeryeong, Noksapyeong, Daecheong, Daeheung, Dogok, Dorimcheon, Dongnimmun (Independence) Gate , Dokbawi , Dongjak, Dunchon-dong, Magok, Majang, Macheon, Mongchon toseong, Muakjae, Banpo, Bangi, Sangwolgok, Seokchon, Songjeong, Singeumho, Singil, Sinnae, Sindap, Sinseoldong, Aeogae, Yaksu, Yangcheon-gu Office, Yangpyeong, Yeokcheon, Yeongdeungpo Market, Ogeum, Olympic Park, Yongdap, Yongdu, Yongmasan, World Cup Stadium, Ichon, Jamwon, Sports Complex, Changsin, Cheonggu, Taereung Entrance, Hangnyeoul , Hanyang University, Haengdang, Hyochang Park
2	Gangnam, Guro Digital Complex, Seoul National University Entrance, Sindorim, Sillim, Hongdae Entrance
3	Gasan Digital Complex, Express Bus Terminal, Gwanghwamun, Nambu Bus Terminal, Samsung, Seoul, Seolleung, Seongsu, City Hall, Sinsa, Sinchon, Apgujeong, Yangjae, Yeouido, Yeoksam, Euljiro Entrance, Jamsil, Jonggak, Hakdong, Hyehwa
4	Gangnam-gu Office, Gyeongbokgung, Gongdeok, Gyodae, Namguro, Naebang, Nonhyeon, Dongdaemun, Dongdaemun History and Culture Park, Dongdaemun Entrance, Dongmyo, Digital Media City, Ttukseom, Mapo, Mangwon, Maebong, Myeongdong, Mullae, Munjeong, Balsan, Bangbae, Sangsu, Seodaemun, Seocho, Suseo, Sookmyung Women’s University, Sinyongsan, Anguk, Anam, Children’s Grand Park, Yeouinaru, Yeongdeungpo-gu Office, Euljiro 3-ga, Eulji-ro 4-ga, Ewha Womans University, Isu, Itaewon, Irwon, Jamsil Naru, Jangji, Jang-han-pyeong, Jongno3-Ga, Jongno 5-ga, Cheongdam, Chungmuro, Hangangjin, Hoehyeon
5	Gangdong, Gangbyeon (Dongseoul Bus Terminal), Konkuk University, Guui, Gupabal, Gireum, Kkachisan, Nakseongdae, Nowon, Dangsan, Daerim, Mokdong, Miasageori,Bongcheon, Sadang, Sanggye, Sangbong, Sangil-dong, Sungshin Women’s University, Suyu, Sindaebang, Ssangmun, Amsa, Yeonsinnae, Omokgyo, Eungam, Jamsil saenae, Jegi-dong, Chang-dong, Cheonho, Cheongnyangni, Chongsin University, Hagye, Hapjeong, Hongje, Hwagok
6	Gangdong-gu Office, Gongneung, Gwangnaru, Gunja, Gubeundari (Gangdong Community Center), Namseong, Nokbeon, Dapsimni, Danggogae, Daechi, Dobongsan, Dolgoch, Ttukseom Resort, Madeul, Mapo-gu Office, Meokgol, Myeonmok, Myeongil, Mia, Bokjeong, Bonghwasan, Bulgwang, Sagajeong, Sangdo, Sangwangsimni, Saejeol, Seokgye, Suraksan, Soongsil University entrance, Sindang, Sindaebangsamgeori, Sinjeong, Sinjeong negeori, Sinpung, Achasan, Ahyeon, Oksu, Onsu, Wangsimni, Ujangsan, Wolgok, Jangseungbaegi, Junggye, Junghwa, Jeungsan, Cheonwang, Hansung University entrance, Hwarang-dae

### Prediction results

**Figure 11:**
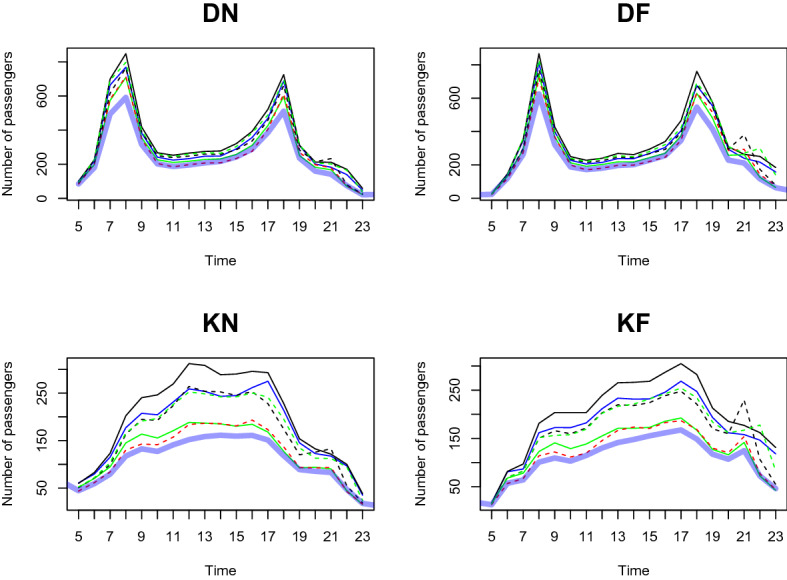
A Comparison of the predicted and actual daily number of passengers for cluster 1.

**Figure 12:**
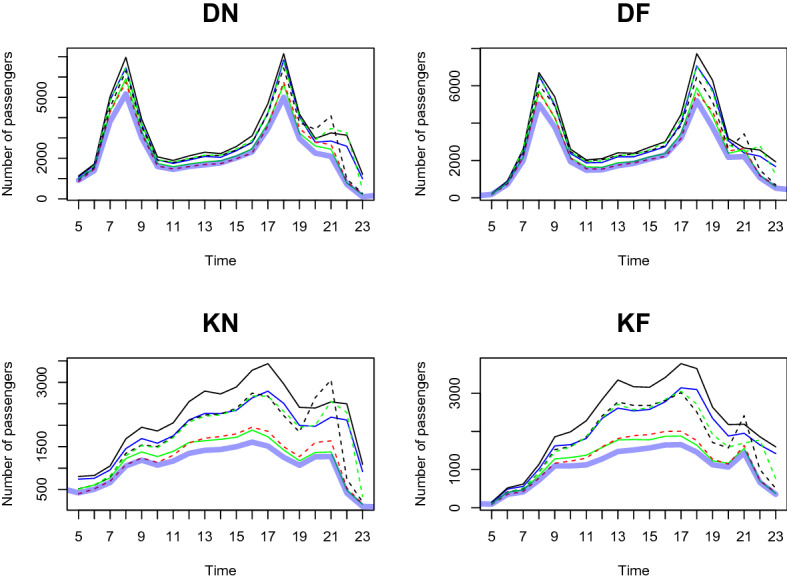
A Comparison of the predicted and actual daily number of passengers for cluster 2.

**Figure 13:**
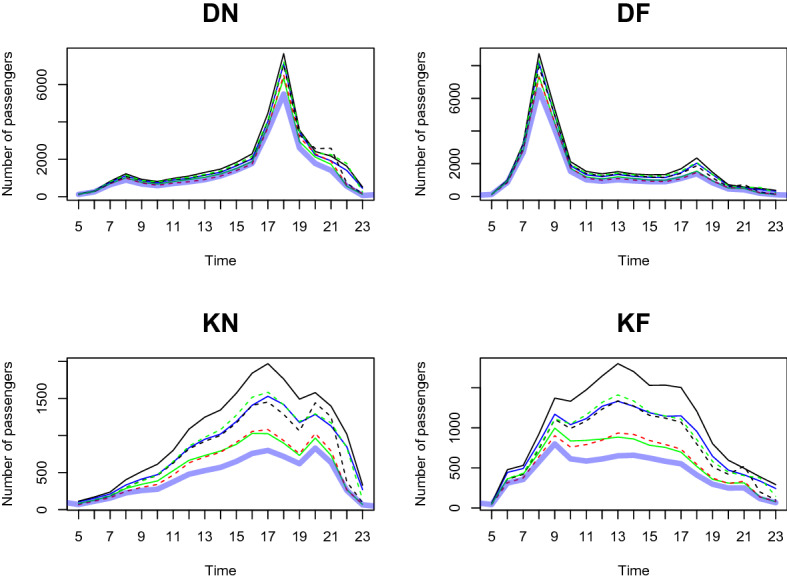
A comparison of the predicted and actual daily number of passengers for cluster 3.

**Figure 14:**
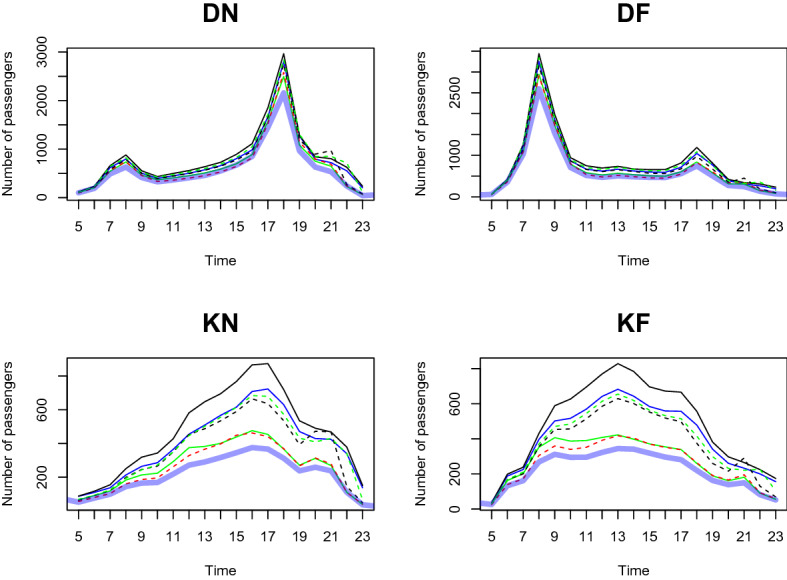
A comparison of the predicted and actual daily number of passengers for cluster 4.

**Figure 15:**
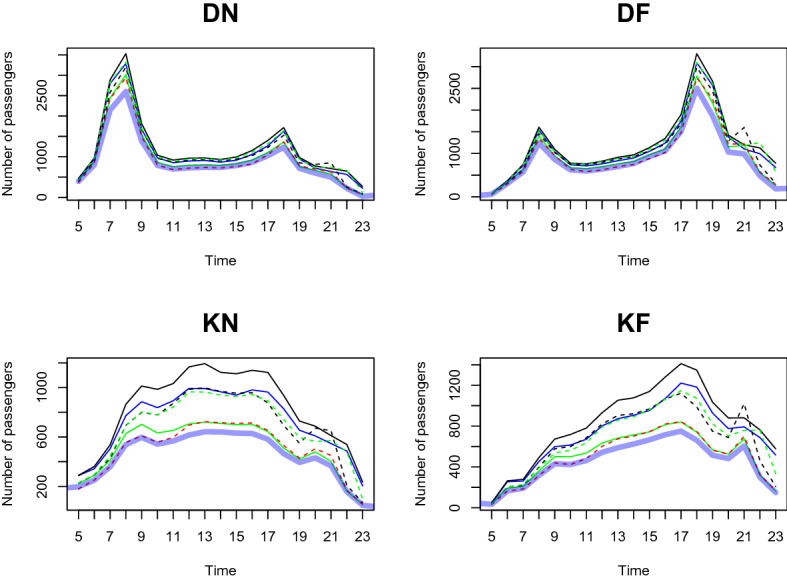
A comparison of the predicted and actual daily number of passengers for cluster 5.

**Figure 16:**
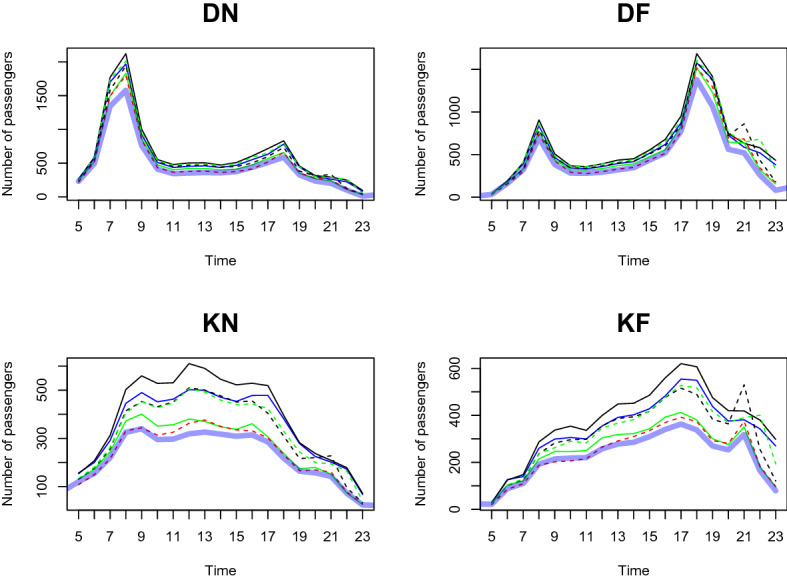
A comparison of the predicted and actual daily number of passengers for cluster 6.

**Figure 17:**
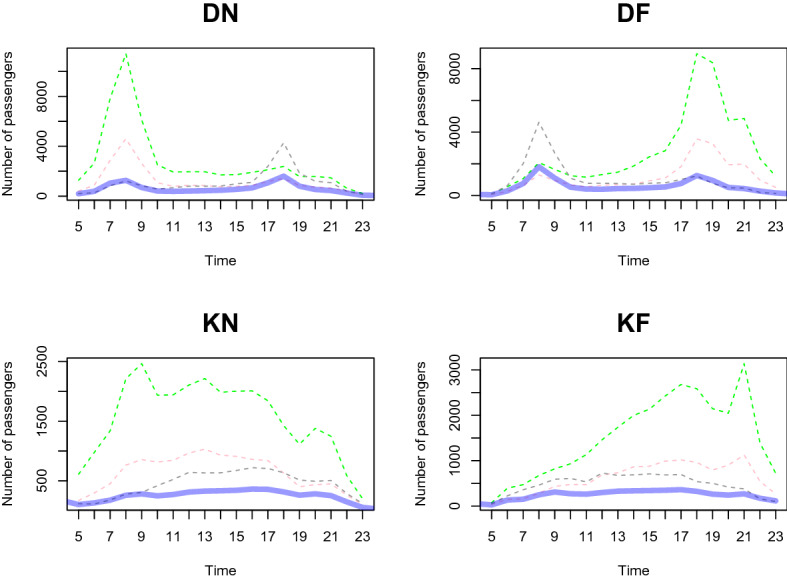
A comparison of the predicted and actual daily number of passengers without considering regional properties.

We predict the daily passenger transport flow for the first week of January 2021 based on the dataset from January 2020 to December 2020, in a 1-h interval. In particular, we choose the first week of January 2021 to demonstrate the prediction accuracy of our two-step method. This is because South Korea controlled Covid-19 well in 2020 and the number of passengers shows a moderate trend throughout the year. However, as alpha variants were transmitted to South Korea at the end of 2020, the government implemented a lockdown from the end of 2020 to early 2021. We want to demonstrate the prediction result based on the geographical information near the station predict the number of and the patterns of passengers well compared to the outcome without clustering analysis. From Figs. 11, 12, 13, 14, 15, 16, blue thick solid lines and red dotted lines are predicted and the actual number of passengers for week 1 of 2021, respectively. Blue, black, and green solid lines are the actual number of passengers for the week 41, 45, and 50 of 2020, respectively. Black and green dotted lines are the actual number of passengers for weeks 5 and 9 of 2021, respectively. As we can see from Figs. 11, 12, 13, 14, 15, 16, there is an increasing trend from 41st to 45th week in 2020 and a decreasing trend from week 45, 2020 to week 1 in 2021. Then, we can see another increasing tendency from week 1 to 9 in 2021. Figures 11, 12, 13, 14, 15, 16 present our two-step prediction method not only accurately predicts the number of passengers for week 1 of 2021 but also well reflects the regional characteristic of each cluster, including the number of passenger flow. For example, predicted functions in cluster 1 reflect a lower number of passengers compared to the other clusters and prediction results in cluster 2 show the characteristic of downtown or transit center areas. Clusters 3 and 4 show the predicted number of passenger flows in the business district as we can see there are sharp peaks of DN in the evening and DF in the morning. In particular, they also can well reflect regional properties that cluster 3 has a larger number of passengers than cluster 4. Furthermore, the prediction outcome based on the clustering results can capture a characteristic of the residential areas, such as a larger number of passengers of DN in the morning and DF in the afternoon, and there is a small hike in the number of people who leave the subway station at the night of the weekends. Figure 17 presents the prediction functions of four situations, DN, DF, KN, KF, based on the averaged data of 2020 without clustering. In Fig. 17, green dotted lines present Sillim station which is one of the stations in cluster 2, red dotted lines are Nakseongdae station in cluster 5, black dotted lines are Seoul station in cluster 3, and thick blue solid lines are the predicted number of passengers without considering regional characteristics. As we can notice from Fig. 17, the prediction functions cannot capture the patterns of the Sillim, Nakseongdae, and Seoul station and there is a discrepancy in the number of passengers. For example, in DN and DF cases, the predicted functions have two peaks. This result is not suitable for the stations in both commercial or residential areas. Therefore, if we want to adjust service intervals to improve passenger ridership or plan to make additional subway stations, using the predicted number of passengers based on the cluster analysis can be helpful as it can provide results reflecting the regional characteristics around the stations.We further present the execution time for prediction in Table 3.

To our best knowledge, our paper is the first research of adopting FDA approach to predict the daily number of Seoul Metro passengers by applying the functional clustering result. Our research has strength in achieving a high prediction accuracy by only utilizing the passenger volume dataset, which can be easily acquired through open source without any additional information about public transportation. Here, we compare our results with^22^ which categorized the stations in Seoul into three clusters and predicted the number of passengers per hour. Cho et al.^22^ chose a model with some extra information and their method was not based on the FDA approach. For the fair comparison, we randomly select three consecutive weeks (weeks 11, 12, 13) and predict the number of passengers of the subsequent week (week 14). Table 4 shows the root mean squared error (RMSE) of our approach with respect to six clusters. Tables 5 and 6 present the averaged RMSE for the prediction of the averaged number of passengers and the maximum number of passengers per hour, respectively, from^22^. As we can see from Tables 4, 5, and 6, our method shows lower total RMSE, which demonstrates its competitiveness.

We use three weeks’ data (training dataset) to train the prediction model and examine the predictive performance with the subsequent one week data (test dataset). We randomly select three weeks (weeks 11, 12, 13 of 2020) as a training dataset and use the data for the very next week (week 14 of 2020) as a test dataset. Figures 19, 20, 21, 22, 23, 24 show the prediction results. Blue thick solid lines and red solid lines are predicted and the actual number of passengers for week 14 of 2020, respectively. As we can see from Figs. 19, 20, 21, 22, 23, 24, our method works well.

**Table 3:** The execution time for prediction in seconds.

Cluster 1	Cluster 2	Cluster 3	Cluster 4	Cluster 5	Cluster 6
0.078	0.064	0.069	0.070	0.066	0.067

**Table 4:** The averaged RMSE of our approach.

Cluster 1	Cluster 2	Cluster 3	Cluster 4	Cluster 5	Cluster 6	Total
4.889	59.347	31.449	17.446	20.488	11.521	145.14

**Table 5:** The averaged RMSE for the averaged number of passengers in^22^.

Cluster 1	Cluster 2	Cluster 3	Total
66	48	32	146

**Table 6:** The averaged RMSE for the maximum number of passengers in^22^.

Cluster 1	Cluster 2	Cluster 3	Total
234	185	115	534

## Discussion

Subway plays a critical role in public transportation for a number of large cities. With the rapid development of data collecting technologies, we can obtain ‘big data’ from the smart card transaction system and utilize this data to track passenger patterns and improve the subway operation system or service quality. However, there has been a lack of efficient methodologies to analyze massive time series dataset. Here, we incorporate the method of functional cluster analysis to functional time series prediction. First, we split the dataset into four situations based on the patterns; that is, the number of passengers who get on the subway station on weekdays and weekends and the number of passengers who get off the subway during the weekdays and weekends. After we explore the basic properties of the dataset using FPCA and covariance analysis, we implement functional clustering by applying the funFEM^19^ and categorize the entire subway stations into six clusters. We present that each cluster can successfully capture its regional characteristics around the stations and passenger transport patterns. Then, based on the passenger flow of 2020, we predict the daily number of passengers for the first week of 2021 for each cluster in a 1-h interval. We show that the prediction accuracy based on our two-step approach outperforms the result without using cluster analysis. The clustering and prediction results can be applied to a number of other areas. For example, the outcomes can be utilized in urban planning as the prediction result can help to locate the future subway stations or transit centers to improve commuting and housing problems. Furthermore, since we can control the service intervals to avoid heavy congestion, our method can be used to relieve the spread of infectious diseases such as Covid-19 and improve the comfortable ridership experience.

For future research, we can apply our two-step approach to vehicle transport flow to relieve a traffic jam in a large city because we can provide a prediction based on the clustering result, which reflects the characteristics of the cities. This research can further be used in predicting the number of Covid-19 cases, considering regional and cultural characteristics.

## Methods

### Functional data analysis

Functional data is one of the most prevalent formats of the dataset in contemporary research, such as anthropology, biology, forensic science, and many others. It has an especially complex and structured format that can be viewed as smooth curves or functions rather than numbers or vectors. Because of its special type of data format, there has been explosive development of a methodology to analyze functional data over the past decade or so. See for example^23–28^.

Let $(\Omega , \mathscr {F}, P)$ be a probability space, $\mathscr {T}\in \mathbb {R}$ an interval, and $L_{\scriptscriptstyle {2}}$ the class of all function of *X* such that $\{ (f: \Omega _{\scriptscriptstyle {X}} \rightarrow \mathbb {R}: \int f dP=0, \int f^{\scriptscriptstyle {2}} dP < \infty \}$. FDA considers each observation as a smooth $L_{\scriptscriptstyle {2}}$ stochastic process which can be denoted as *X*(*t*) with $t\in \mathscr {T}$. However, there is a generic limitation that we can only obtain a finite and discrete number of the dataset; that is, the provided data can be represented as $X(t_{\scriptscriptstyle {j}})$ with $j=\{1, \ldots , m \}$ where *m* is the number of time points for discrete observations. Therefore, to analyze the dataset in FDA contexts, we are required to express the discrete observations in a functional format. One of the most widely used methods is based on the assumption that smooth curves are approximated using the finite-dimensional subspace spanned by the basis functions. This can be represented as

1 $$\begin{aligned} X_{\scriptscriptstyle {i}}(t) = \sum _{\scriptscriptstyle {j=1}}^{\scriptscriptstyle {M}} C_{\scriptscriptstyle {ij}} B_{\scriptscriptstyle {j}}(t), \end{aligned}$$

where $B_{\scriptscriptstyle {j}}(t)$ is the basis functions such as B-spline or Fourier basis, $C_{\scriptscriptstyle {ij}}$ is a coefficient, and *M* is the number of basis functions. The coefficients can be determined by using interpolation^29^, least square, or penalized least square methods. For more details of the fundamentals of FDA, see^23,24^, and^30^.

### Functional principal component analysis

After we obtain a functional expression of (1) from the original dataset, we can express the mean and covariance in a functional format as

$$\begin{aligned} \mu (t)&= E[X_{\scriptscriptstyle {n}}(t)], \\ C(t,s)&= E[(X_{\scriptscriptstyle {n}}(t) - \mu (t)) {(X_{\scriptscriptstyle {n}}(s) - \mu (s)) }]. \end{aligned}$$

Similar to their multivariate counterpart, the mean is the pointwise average and the covariance indicates pointwise variability of curves. The sample version of the mean and covariance can be represented as

$$\begin{aligned} \hat{\mu }(t)&= \frac{1}{N} \sum _{\scriptscriptstyle {n=1}}^{\scriptscriptstyle {N}} X_{\scriptscriptstyle {n}}(t) = E_{\scriptscriptstyle {n}}[X_{\scriptscriptstyle {n}}(t)], \\ \hat{C}(t,s)&= E_{\scriptscriptstyle {n}}[(X_{\scriptscriptstyle {n}}(t) - \hat{\mu }(t)) {(X_{\scriptscriptstyle {n}}(s) - \hat{\mu }(s)) }]. \end{aligned}$$

Functional principal component analysis (FPCA) is one of the most widely used dimension reduction methods in FDA. The problem of FPCA can be represented as finding the first few eigenfunctions from the covariance function which is formulated as finding $e_{\scriptscriptstyle {1}}, \ldots , e_{\scriptscriptstyle {d}}$ satisfying

$$\begin{aligned} \lambda _{\scriptscriptstyle {i}} e_{\scriptscriptstyle {i}} = \int _{\scriptscriptstyle {\mathscr {T}}} C(t,s) e_{\scriptscriptstyle {i}}(s) ds \quad \text {with} \quad \int e_{\scriptscriptstyle {i}}(t)^{\scriptscriptstyle {2}} dt = 1, \end{aligned}$$

where $\mathscr {T}$ is an interval. The result of FPCA represents the most important shape of variability deviated from the mean function. The sample version of FPCA can be presented as

$$\begin{aligned} \hat{\lambda }_{\scriptscriptstyle {i}} \hat{e}_{\scriptscriptstyle {i}} = \int _{\scriptscriptstyle {\mathscr {T}}} \hat{C}(t,s) \hat{e}_{\scriptscriptstyle {i}}(s) ds \quad \text {with} \quad \int \hat{e}_{\scriptscriptstyle {i}}(t)^{\scriptscriptstyle {2}} dt = 1, \end{aligned}$$

where $\mathscr {T}$ is again an interval. Infinite dimensional functional data can be expressed using a reasonably small number of functional principal components as

$$\begin{aligned} X_{\scriptscriptstyle {n}}(t) - \overline{X}(t) \approx \sum _{\scriptscriptstyle {i=1}}^{\scriptscriptstyle {p}} \hat{\varepsilon }_{\scriptscriptstyle {ni}} \hat{e}_{\scriptscriptstyle {i}}(t), \end{aligned}$$

where *p* is much smaller than *M* in (1) and

$$\begin{aligned} \hat{\epsilon }_{\scriptscriptstyle {ni}} = \int (X_{\scriptscriptstyle {n}}(t) - \overline{X}(t)) \hat{e}_{\scriptscriptstyle {i}}(t) dt. \end{aligned}$$

$\hat{\epsilon }_{\scriptscriptstyle {ni}}$ is called the principal component scores and can be widely used in the FDA modeling process such as functional clustering and prediction. The dimension reduction is facilitated by determining a finite number *p* such that the sum of the first *p* terms provides a good approximation to the infinite sum. The selection of *p* is usually done through cross-validation. See^31^ for more details.

### Functional clustering

The method of functional clustering algorithm allows categorizing time series or, more generally, functional data. Various functional clustering methods have been developed in the past decades. Abraham et al.^32^ applied K-means clustering and Rossi et al.^33^ used a neural network to the coefficients after B-spline basis expansion. Peng and Müller^34^ used the K-means method to the principal component scores. Furthermore, some methodologies use a fully nonparametric approach combining the specific measure of distance to K-means clustering^35–37^ or hierarchical clustering^26^. There were also model-based approaches which hinges on functional principal component scores^19,38–40^ and the coefficient from the basis expansion^41–45^. For a more comprehensive review, see^46^. In particular, we use a recently developed model-based functional clustering method called the funFEM^47^. This model has its own strength in sparsity which can make it possible to handle the long time series data. Here, we introduce the basic principles of the funFEM algorithm, which hinges on a discriminative functional mixture model. This method allows the clustering of the data in a unique and discriminative functional subspace.

Suppose we want to separate our dataset into *K* clusters where *K* is a fixed integer. The clusters can be regarded as unobserved variables and can be denoted as $Y=\{Y_{\scriptscriptstyle {1}}, \ldots , Y_{\scriptscriptstyle {K}}\} \in \mathbb {R}^{\scriptscriptstyle {K}}$ with each element is from $\{0, 1\}$. Then, we assume there is a latent subspace of $L_{\scriptscriptstyle {2}}$ which can be the most discriminative to divide the dataset in *K* clusters and this latent subspace is spanned by $B_{\scriptscriptstyle {1}}^{\scriptscriptstyle {\prime }}(t) , \ldots , B_{\scriptscriptstyle {q}}^{\scriptscriptstyle {\prime }}(t)$ where *q* is smaller than *M* in (1). Similar to (1), we can approximate observed dataset as

2 $$\begin{aligned} X_{\scriptscriptstyle {i}}(t) = \sum _{\scriptscriptstyle {j=1}}^{\scriptscriptstyle {q}} G_{\scriptscriptstyle {ij}} B_{\scriptscriptstyle {j}}^{\scriptscriptstyle {\prime }}(t). \end{aligned}$$

Then, we can represent the relationship between the basis $B_{\scriptscriptstyle {j}}(t)$ in (1) and the basis of latent subspaces as

3 $$\begin{aligned} B_{\scriptscriptstyle {a}}^{\scriptscriptstyle {\prime }}(t) = \sum _{\scriptscriptstyle {i=1}}^{\scriptscriptstyle {p}} D_{\scriptscriptstyle {ai}} B_{\scriptscriptstyle {i}}(t). \end{aligned}$$

Let *D* be a $p \times q$ orthogonal matrix, G a *q* dimensional coefficient vector of (2), and *C* a *p* dimensional coefficient vector from the basis expansion based on (1) as

$$\begin{aligned} G&=\left( \begin{array}{c} G_{\scriptscriptstyle {i1}} \\ \vdots \\ G_{\scriptscriptstyle {iq}} \end{array} \right) , \quad C =\left( \begin{array}{c} C_{\scriptscriptstyle {i1}} \\ \vdots \\ C_{\scriptscriptstyle {ip}} \end{array} \right) , \\ D&=\left( \begin{array}{ccc} \begin{array}{l} D_{\scriptscriptstyle {11}} \end{array}&\cdots&\begin{array}{l} D_{\scriptscriptstyle {q1}} \end{array} \\ \vdots &{} \ddots &{} \vdots \\ \begin{array}{l} D_{\scriptscriptstyle {1p}} \end{array} &{} \cdots &{} \begin{array}{l} D_{\scriptscriptstyle {qp}}\\ \end{array} \end{array} \right) . \end{aligned}$$

Then, from Eqs. (1), (2), and (3), we represent the relationship between $C_{\scriptscriptstyle {ij}}, G_{\scriptscriptstyle {ij}}$ and $D_{\scriptscriptstyle {ai}}$ in a matrix form as

$$\begin{aligned} C = DG + \epsilon , \end{aligned}$$

where $\epsilon$ is a *p* dimensional independent noise. Bouveyron et al.^47^ assumes that in the *k*-th cluster, *G* follows multivariate Gaussian distribution which can be represented as

$$\begin{aligned} G^{\scriptscriptstyle {k}} \sim N( \mu _{\scriptscriptstyle {k}}, \Sigma _{\scriptscriptstyle {k}} ), \end{aligned}$$

where $G^{\scriptscriptstyle {k}}$ is *G* in the *k*-th cluster, $\mu _{\scriptscriptstyle {k}}, \Sigma _{\scriptscriptstyle {k}}$ are a mean vector and a covariance matrix, respectively. Furthermore, $\epsilon$ is also assumed to be from the centered Gaussian distribution with $\Sigma _{\scriptscriptstyle {\epsilon }}$ as a covariance matrix. Then the marginal distribution of *C* in the *k*-th cluster can be represented as

$$\begin{aligned} P(Z=k) g(D \mu _{\scriptscriptstyle {k}}, D^{{\tiny \mathsf{T}}}\Sigma _{\scriptscriptstyle {k}} D + \Sigma _{\scriptscriptstyle {\epsilon }}), \end{aligned}$$

where $P(Z=k)$ is a prior of the *k*-th group and *g* is the Gaussian density function. Here, because *D* is from the latent subspace of $L_{\scriptscriptstyle {2}}$ which makes the clustering most discriminative, we need to estimate *D* first and implement an Expectation Maximization (EM) algorithm^48^. The step that estimates *D* is called an *F* step and therefore, this whole model-based clustering method is called the funFEM.

### Functional time series forecast

**Figure 18:**
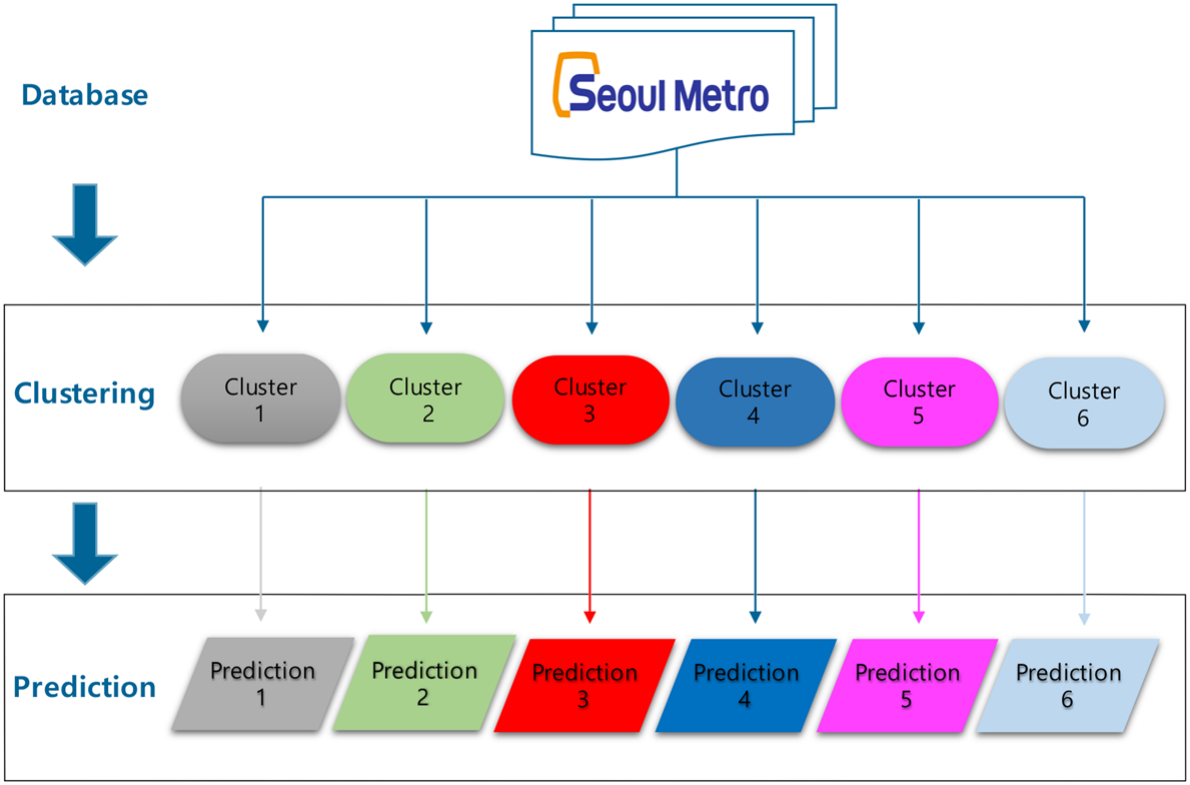
A diagram of a two-step procedure.

**Figure 19:**
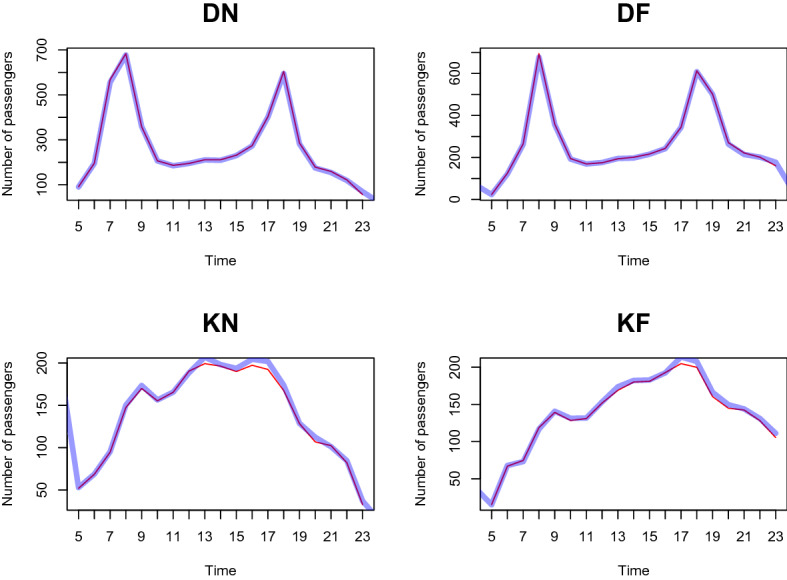
A comparison of the predicted and actual daily number of passengers for cluster 1 based on the test dataset.

**Figure 20:**
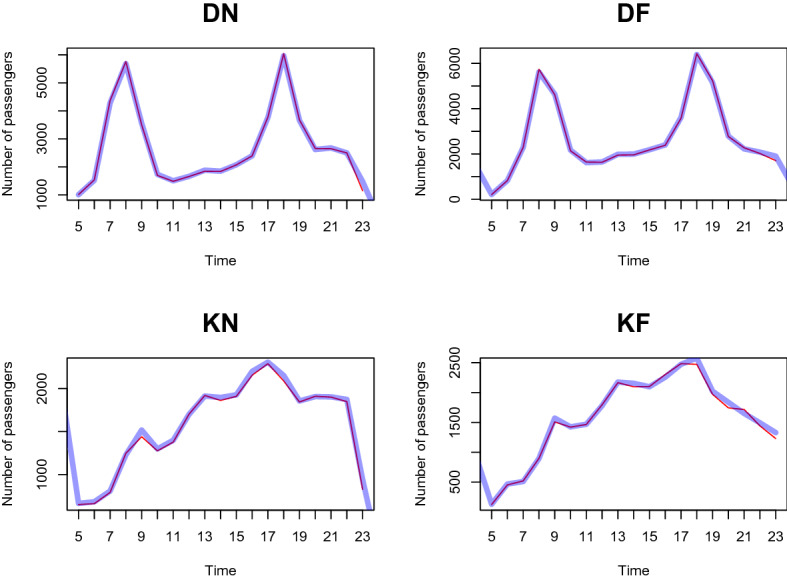
A comparison of the predicted and actual daily number of passengers for cluster 2 based on the test dataset.

**Figure 21:**
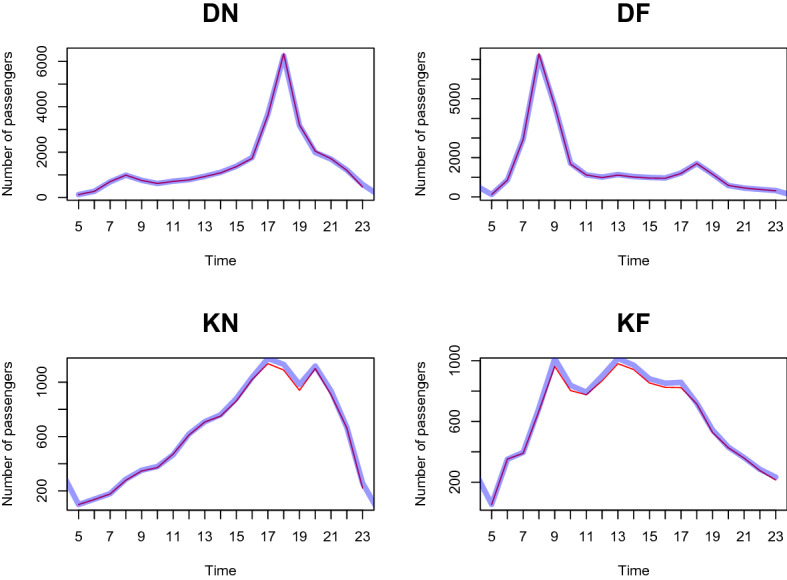
A comparison of the predicted and actual daily number of passengers for cluster 3 based on the test dataset.

**Figure 22:**
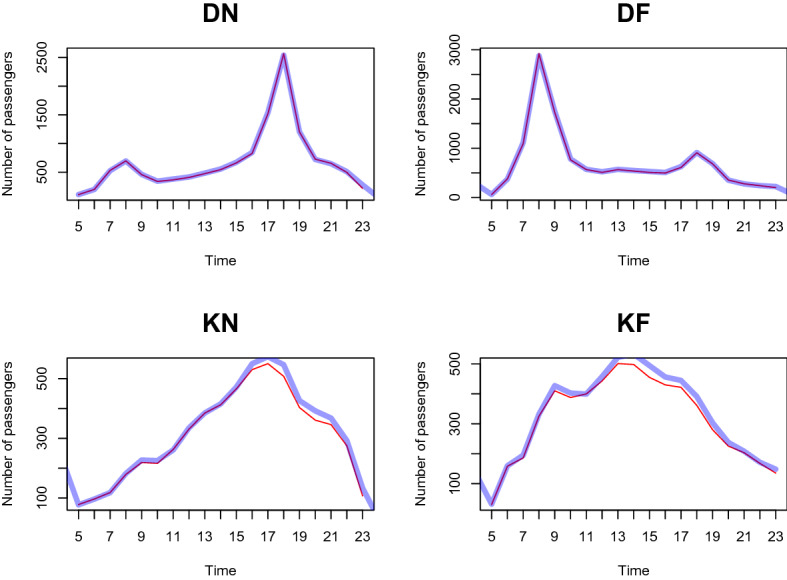
A comparison of the predicted and actual daily number of passengers for cluster 4 based on the test dataset.

**Figure 23:**
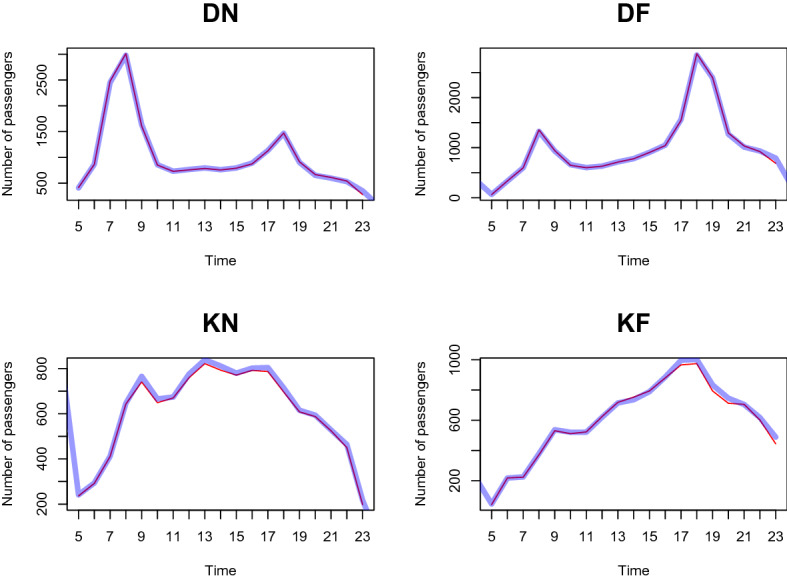
A comparison of the predicted and actual daily number of passengers for cluster 5 based on the test dataset.

**Figure 24:**
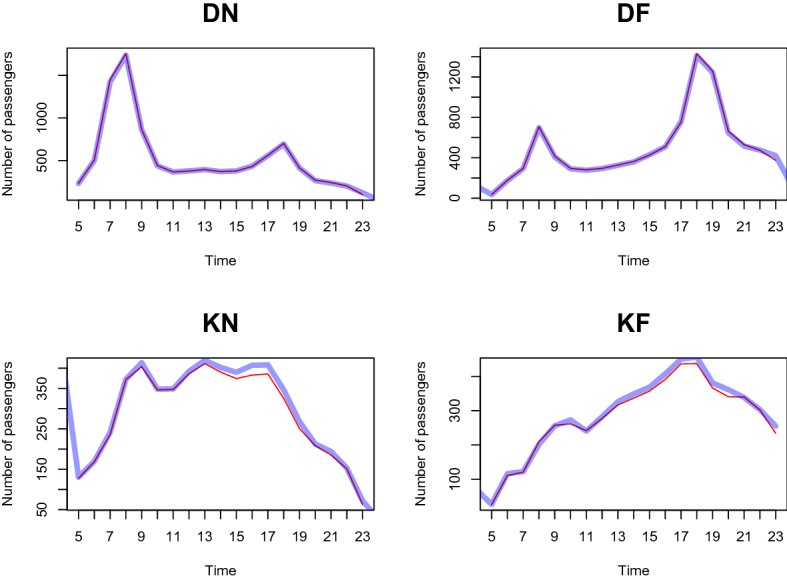
A comparison of the predicted and actual daily number of passengers for cluster 6 based on the test dataset.

As we can see from Fig. 18, another main point of our research is that we predict the number of passengers based on the regional information from the functional clustering method. Thus, it is essential to describe a procedure to predict the number of people on the subway using a functional time series. The main strategy of functional time series prediction is using the scores from FPCA as a module and using them for time series forecasting. Similar to functional clustering, first of all, we smooth the dataset using a Fourier basis to express the dataset in a curve. Then we apply FPCA to decompose the smoothed curve in scores and principal components. The next step is fitting and forecasting each score using a times series model such as ARIMA, AR, or exponential smoothing model. Finally, we recover the result into a functional data form by multiplying the predicted scores to the principal components.

## Appendix

See Figs. 19, 20, 21, 22, 23, 24.

## References

[1] Tang T (2018). Fiss: Function identification of subway stations based on semantics mining and functional clustering. IET Intell. Transp. Syst..

[2] Wang J (2017). Is2fun: Identification of subway station functions using massive urban data. IEEE Access.

[3] Ling X, Huang Z, Wang C, Zhang F, Wang P (2018). Predicting subway passenger flows under different traffic conditions. PLoS ONE.

[4] Kim K-Y, Lim C-Y, Kim EJ (2018). A new approach to the space-time analysis of big data: Application to subway traffic data in Seoul. J. Big Data.

[5] Yu W, Bai H, Chen J, Yan X (2019). Analysis of space-time variation of passenger flow and commuting characteristics of residents using smart card data of nanjing metro. Sustainability.

[6] Shin H (2020). Analysis of subway passenger flow for a smarter city: Knowledge extraction from Seoul metro’s ‘untraceable’ big data. IEEE Access.

[7] Liu S, Yao E (2017). Holiday passenger flow forecasting based on the modified least-square support vector machine for the metro system. J. Transp. Eng. Part A Syst..

[8] Chen C, Chen J, Barry J (2009). Diurnal pattern of transit ridership: A case study of the New York city subway system. J. Transp. Geogr..

[9] Alan UD, Birant D (2018). Server-based intelligent public transportation system with NFC. IEEE Intell. Transp. Syst. Mag..

[10] Pelletier M-P, Trépanier M, Morency C (2011). Smart card data use in public transit: A literature review. Transp. Res. Part C Emerg. Technol..

[11] Lim H-J (2005). A study on transit-oriented development method to activate transit use for high urban-density muti-nucleated Seoul. J. Korean Soc. Transp..

[12] Oh, J., Hong, S.-Y. & Jin, J. The relationship between planning elements of 5Ds RTOD and transit ridership: A focus on job accessibility in Seoul. *J. Korean Geogr. Soc.* **54**(6), 609–620 (2019).

[13] Sohn D, Kim J (2010). Analysis of the relationships between land use characteristics of urban transit centers and the level of transit usage: Case studies of seoul metropolitan area. J. Urban Desi. Inst. Korea.

[14] Lee J-A, Cho M-S, Koo J-H (2013). Relationship between mixed land-use characteristics and time-based patterns of subway users: Focused on the surrounding areas of seoul subway stations. J. Korea Plan. Assoc..

[15] Kim S, Eom S, Lee M (2013). A study on spatial range of Seoul subway station area on characteristics of land use. J. Korea Plan. Assoc..

[16] Sung H-G, Kim T-H (2005). A study on categorizing subway station areas in Seoul by rail use pattern. J. Korean Soc. Transp..

[17] Choi H-S, Kim T-H, Lee J-H (2013). A study on the classification of the spatial characteristics by TOD planning elements of subway station areas in Seoul. J. Korean Assoc. Geogr. Inf. Stud..

[18] Lee K-S, Song Y-N, Park J-S, Anderson WP (2012). Relationship between diurnal patterns of transit ridership and land use in the metropolitan Seoul area. J. Econ. Geogr. Soc. Korea.

[19] Bouveyron C, Jacques J (2011). Model-based clustering of time series in group-specific functional subspaces. Adv. Data Anal. Classif..

[20] Schmutz A, Jacques J, Bouveyron C, Cheze L, Martin P (2020). Clustering multivariate functional data in group-specific functional subspaces. Comput. Stat..

[21] R Core Team (2020). R: A Language and Environment for Statistical Computing.

[22] Cho S, Kim B, Kim N, Song J (2019). A study on the number of passengers using the subway stations in Seoul. Korean J. Appl. Stat..

[23] Ramsay JO, Silverman BW (2005). Functional Data Analysis.

[24] Ramsay JO, Silverman BW (2007). Applied Functional Data Analysis: Methods and Case Studies.

[25] Yao F, Müller H-G, Wang J-L (2005). Functional data analysis for sparse longitudinal data. J. Am. Stat. Assoc..

[26] Ferraty F, Vieu P (2006). Nonparametric Functional Data Analysis: Theory and Practice.

[27] Horváth L, Kokoszka P (2012). Inference for Functional Data with Applications.

[28] Hsing T, Eubank R (2015). Theoretical Foundations of Functional Data Analysis, with an Introduction to Linear Operators.

[29] Escabias M, Aguilera A, Valderrama M (2005). Modeling environmental data by functional principal component logistic regression. Environmetrics.

[30] Kokoszka P, Reimherr M (2017). Introduction to Functional Data Analysis.

[31] Boeing, P. & Wang, Y. Decoding China’s covid-19 ‘virus exceptionalism’: Community-based digital contact tracing in Wuhan. *R&D Manag.* **51**(4), 339–351 (2021).

[32] Abraham C, Cornillon P-A, Matzner-Løber E, Molinari N (2003). Unsupervised curve clustering using b-splines. Scand. J. Stat..

[33] Rossi, F., Conan-Guez, B. & El Golli, A. Clustering functional data with the som algorithm. In *ESANN*, 305–312 (Citeseer, 2004).

[34] Peng J, Müller H-G (2008). Distance-based clustering of sparsely observed stochastic processes, with applications to online auctions. Ann. Appl. Stat..

[35] Ieva, F., Paganoni, A. M., Pigoli, D. & Vitelli, V. Multivariate functional clustering for the analysis of ecg curves morphology. In *Cladag 2011 (8th International Meeting of the Classification and Data Analysis Group)*, 1–4 (2011).

[36] Tarpey, T. & Kinateder, K. K. Clustering functional data. *J. Classif.* **20**, 93–114 (2003).

[37] Tipping ME, Bishop CM (1999). Mixtures of probabilistic principal component analyzers. Neural Comput..

[38] Chiou J-M, Li P-L (2007). Functional clustering and identifying substructures of longitudinal data. J. R. Stat. Soc. Ser. B Stat. Methodol..

[39] Jacques J, Preda C (2013). Funclust: A curves clustering method using functional random variables density approximation. Neurocomputing.

[40] Jacques J, Preda C (2014). Model-based clustering for multivariate functional data. Comput. Stat. Data Anal..

[41] Giacofci M, Lambert-Lacroix S, Marot G, Picard F (2013). Wavelet-based clustering for mixed-effects functional models in high dimension. Biometrics.

[42] Heard NA, Holmes CC, Stephens DA (2006). A quantitative study of gene regulation involved in the immune response of anopheline mosquitoes: An application of bayesian hierarchical clustering of curves. J. Am. Stat. Assoc..

[43] James GM, Sugar CA (2003). Clustering for sparsely sampled functional data. J. Am. Stat. Assoc..

[44] Ray S, Mallick B (2006). Functional clustering by Bayesian wavelet methods. J. R. Stat. Soc. Ser. B Stat. Methodol..

[45] Samé A, Chamroukhi F, Govaert G, Aknin P (2011). Model-based clustering and segmentation of time series with changes in regime. Adv. Data Anal. Classif..

[46] Jacques J, Preda C (2014). Functional data clustering: A survey. Adv. Data Anal. Classif..

[47] Bouveyron C, Côme E, Jacques J (2015). The discriminative functional mixture model for a comparative analysis of bike sharing systems. Ann. Appl. Stat..

[48] Dempster AP, Laird NM, Rubin DB (1977). Maximum likelihood from incomplete data via the EM algorithm. J. R. Stat. Soc. Ser. B Stat. Methodol..

